# MDCT cystography using vaginal tampon for imaging lower urogenital tract fistulas: two case reports

**DOI:** 10.1259/bjrcr.20150288

**Published:** 2016-02-06

**Authors:** Martin Ian Kamanda

**Affiliations:** Radiology Department, M.P. Shah Hospital, Nairobi, Kenya

## Abstract

Multidetector CT (MDCT) cystography combines the established technique of excretory cystography with the high spatial resolution of MDCT. MDCT cystography with the use of vaginal tampon helps in increasing the sensitivity and specificity of detecting the presence or absence of urogenital fistulas. Vaginal tampons act as both negative and positive contrast agents. Until recently, urogenital fistulas were investigated by excretory urography/intravenous urography, retrograde ureterography, or retrograde voiding cystography/micturating urography. Scintigraphy with ^99m^Tc diethylenetriaminepentaacetic acid has also been used to help elucidate these pathologic abnormalities. All these modalities are useful in the detection of obvious fistulous tracts but are deficient in providing anatomic detail and have a low specificity.

## Summary

Multidetector CT (MDCT) cystography combines the established technique of excretory cystography with the high spatial resolution of MDCT. MDCT cystography with the use of vaginal tampon helps in increasing the sensitivity and specificity of detecting the presence or absence of urogenital fistulas. Vaginal tampons act as both negative and positive contrast agents.

Until recently, urogenital fistulas were investigated by excretory/intravenous urography, retrograde ureterography or retrograde voiding cystography/micturating urography. Scintigraphy with ^99m^Tc diethylenetriaminepentaacetic acid has also been used to help elucidate these pathological abnormalities.^1^ All these modalities are useful in the detection of obvious fistulous tracts but are deficient in providing anatomic detail and have a low specificity.

## Clinical presentation

### Case 1

A 29-year-old female was recently diagnosed with cancer of the cervix. She had a total hysterectomy and was scheduled for postoperative radiotherapy and chemotherapy. However, she developed urinary incontinence that led to cancellation of both the radiotherapy and chemotherapy sessions. An abdominopelvic CT scan was ordered to check for residual disease and confirm the cause of urinary incontinence.

### Case 2

A 51-year-old female was referred for a CT scan of the abdomen with a clinical suspicion of vesicovaginal fistula (VVF). The patient was diagnosed with cancer of the cervix a year ago and underwent total hysterectomy in the following month. Thereafter, she underwent radiotherapy and chemotherapy. A follow-up CT scan after 11 months showed extensive post-radiation thickening of the pelvic fat planes, bladder walls, vagina and rectosigmoid colon. No enhancing mass lesion, adenopathy, metastatic nodule or other definite evidence of residual tumour was demonstrated. 1 year later, she developed stress incontinence and dripping of urine.

## CT protocol/imaging findings

Permission to write the case reports was sought and granted by the institution through the head of department subject to the author ensuring the anonymity and confidentiality of the patients under study. This was after numerous attempts to obtain consent from the patients or proxies were not forthcoming.

Both the patients were given two Dulcolax tablets to take the previous night. This was carried out to ensure that the bowel loops were free from any faecal matter. On the morning of the examination, the patients were instructed to insert a fresh vaginal tampon. A pre-contrast scan (Somatom Definition AS, 128 slice Siemens, Erlangen, Germany) of the pelvic area was carried out. Using an 18-gauge scalp vein cannula, 30 ml of contrast media (Ultravist 370) was administered intravenously. Delayed scans from the kidneys to the bladder were performed after 8  min.

**Figure 1.**
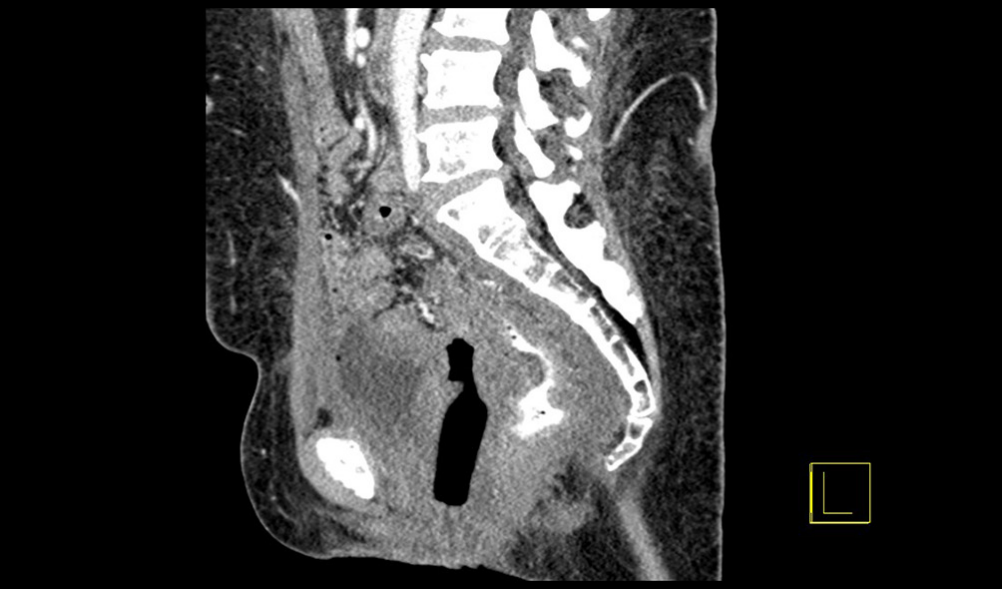
Pre-intravenous contrast CT scan (sagittal reformat) showing the vaginal tampon *in situ* within the vagina for case 1.

In our protocol, the use of vaginal tampons provided both a positive and negative contrast. In the pre-contrast scans, the vaginal tampon acted as a negative contrast agent as it has a low attenuation relative to the pelvic structures (Figure 1). This provided a good differentiation of the cervix or vagina in relation to other pelvic structures. It also allowed reliable localization of the vagina, vaginal cuff and cervix.^2^

**Figure 2.**
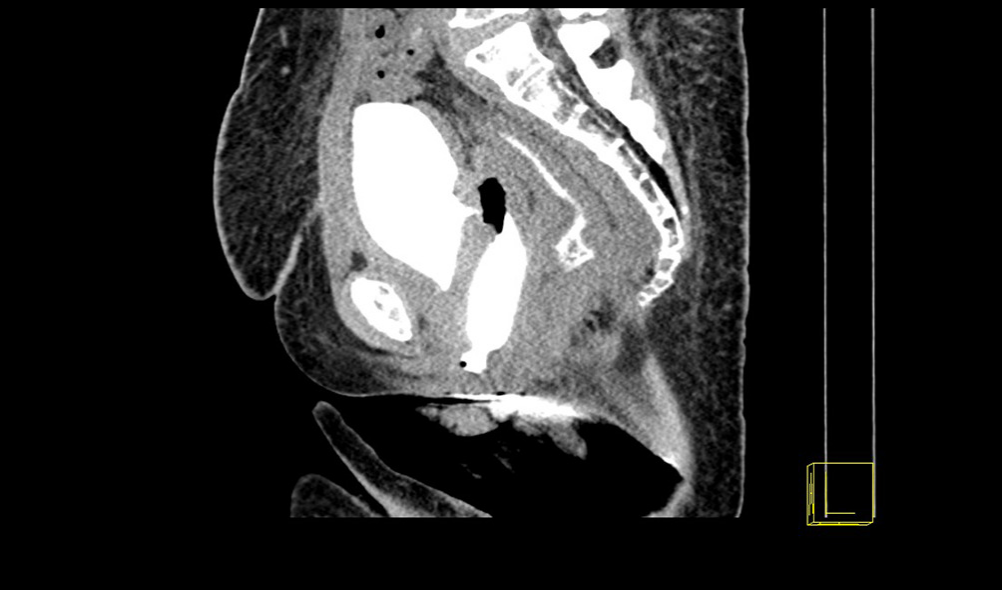
Delayed post-intravenous contrast CT image (sagittal reformat) showing the opacification of the vaginal tampon and the presence of a fistulous tract between the posterior bladder wall and the vagina for case 1.

**Figure 3.**
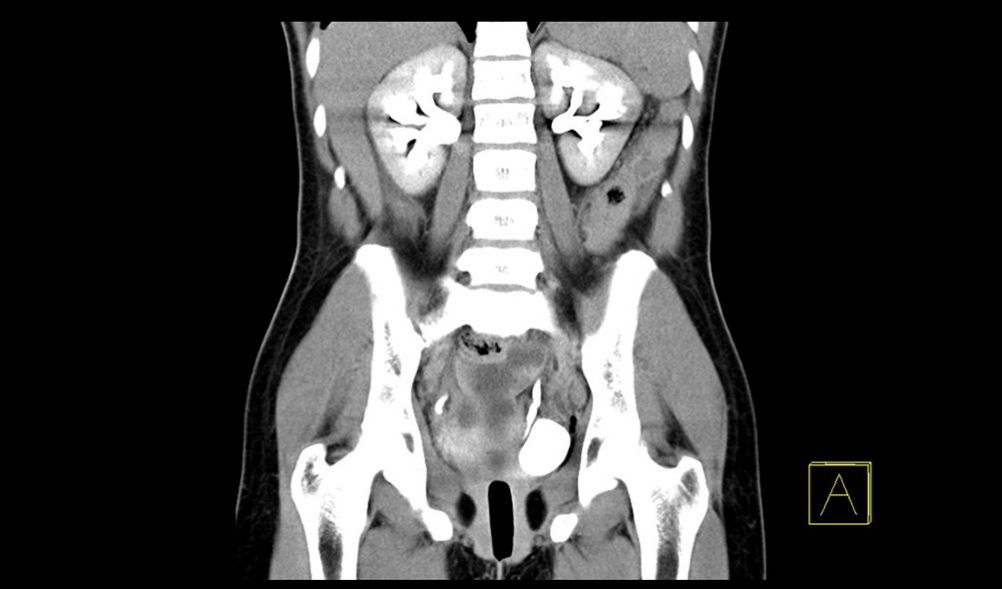
Delayed post-intravenous contrast CT scan (coronal reformat) showing the presence of a hypodense vaginal tampon for case 2.

**Figure 4.**
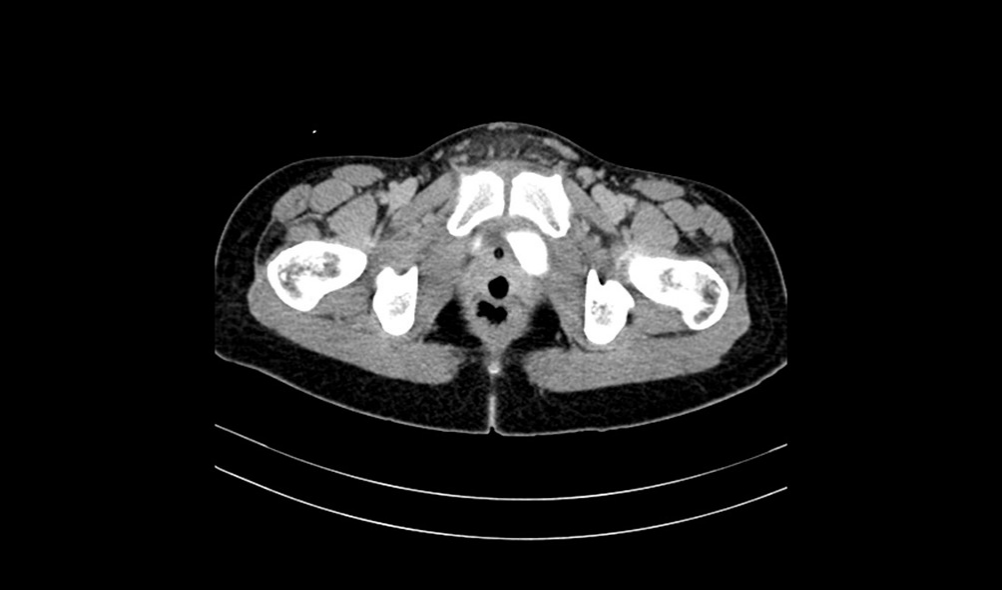
Delayed post-intravenous contrast CT scan (axial reformat) showing the presence of a hypodense vaginal tampon and the absence of a ureterovaginal fistula for case 2.

On the excretory phase of MDCT, in the presence of a fistula, the vaginal tampon soaked with contrast media became hyperdense, thus acting as a positive contrast agent (Figure 2). In the absence of any fistulous communication, the vaginal tampon remained hypodense (Figures 3 and 4). Hence, the course of the fistulous tract in VVF (case 1) and the absence of an ureterovaginal fistula (case 2) were clearly demonstrated. Vaginal tampons in case 1 and 2 helped in improving the sensitivity and specificity, respectively, of MDCT cystography.

## Discussion

The common urogenital fistulas are usually ureterovaginal and vesicovaginal. The uncommon varieties include ureterocutaneous and urethrovaginal fistulas, and fistulous communications between the genitourinary and gastrointestinal tracts. The main causes of urogenital fistulas are obstetrical, surgical, radiation necrosis or malignancy. For patients with known pelvic malignancy, radiation therapy is the common cause of fistulation. Radiotherapy may potentiate fistula formation by inducing progressive endarteritis obliterans. This may result in necrosis and breakdown of the mucosal surfaces, leading to fistulation.^3^

VVF and ureterovaginal fistulas are serious complications that require urgent diagnosis. They often develop as sequelae of surgery or radiation therapy.^4^ There is a propensity for a fistulous communication to form between any diseased or devascularized pelvic and an adjacent organ. This is exacerbated more by recurrent radiation therapy. The unique nature of each fistula type defies a convenient algorithmic approach. However, for the appropriate management of urogenital fistulas, the imaging modality chosen must detect the presence or absence of a fistula, the type of fistula and whether the fistula is simple or complex. In addition, the exact anatomy and trajectory of these fistulas are vital for optimal future management of the condition.^5^

The commonly used imaging modalities in the evaluation of urogenital injuries include excretory urography, renal scintigraphy, CT scan and MRI. Excretory urography has a reported sensitivity of 33% and is usually insufficient to delineate the anatomic details of fistula.^4^ Renal scintigraphy has a high sensitivity in the detection of urinary leaks. However, accurate localization of the fistula site is a major limitation of this modality. All these modalities are useful in the detection of flagrant fistulas but they all lack anatomic detail.^1^

Ultrasonography has been suggested as a valuable alternative that is non-invasive and uses non-ionizing ultrasonic waves for diagnosing the fistulas. However, its role has been limited to the detection of vesicouterine fistulas, permitting correct diagnosis and obviating the need for further examination. Compared with other diagnostic modalities, an MRI is preferable for both disease staging and detection of complications such as a fistula formation. Compared with MDCT, an MRI has a superior soft tissue differentiation that permits the depiction of recurrent tumour, radiotherapy-induced pelvic fibrosis and adhesions within the pelvis.^5^ Currently, in our setting, the cost and inaccessibility is the most inhibiting factor as a modality of choice.

MDCT cystography provides images with exquisite spatial resolution and is readily available. In addition, a CT scan provides the added advantage of reconstructing multiple reformats (axial, coronal, sagittal or three-dimensional) from raw images. This allows better depiction of the fistula site and delineation of the anatomy.^3^

The use of vaginal tampon with MDCT cystography helps in the detection and depiction of VVF and ureterovaginal fistulas. Vaginal tampons act as a negative contrast agent on pre-contrast scans or in the absence of fistulation after intravenous contrast. On post-contrast delayed scans, the vaginal tampon, in the presence of fistulation, acts as positive contrast agent. The vaginal tampon soaks up the excreted contrast media to become hyperdense. In both instances, the vaginal tampon helps in improving the sensitivity and specificity of MDCT cystography in patients suspected to have VVFs and ureterovaginal fistulas.

## Learning points

1. MDCT cystography using vaginal tampons is a simple, inexpensive and safe method to localize the presence, absence and location of lower urinary tract fistulas.
2. MDCT cystography using vaginal tampons is cost-effective and efficient in the absence or unavailability of MRI services.
3. It is also suitable for patients with contraindications to MRI or suffering from claustrophobia.
